# Development of a Strain Sensor Matrix on Mobilized Flexible Substrate for the Imaging of Wind Pressure Distribution

**DOI:** 10.3390/mi11020232

**Published:** 2020-02-24

**Authors:** Shusuke Kanazawa, Hirobumi Ushijima

**Affiliations:** Human Augmentation Research Center, National Institute of Advanced Industrial Science and Technology, 6-2-3 Kashiwanoha, Kashiwa 277-0882, Japan; h-ushijima@aist.go.jp

**Keywords:** flexible electronics, sensors, screen printing, laser cutting, wind pressure

## Abstract

This paper presents a novel flexible sensor for monitoring wind pressure distribution. Based on the concept of “flexible mechatronics”, a suspended structure was incorporated into the matrix of a resistive-strain sensor in a plastic film to make the sensor mechanically movable against the wind. Screen printing and laser cutting were confirmed to be satisfactory methods for fabricating the proposed device structure. As a result, the visualization of wind pressure was successfully demonstrated by the fabricated sensor sheet and an imaging-display-creation software. The results of this study show that a mechanically functionalized substrate opens up new avenues for flexible electronics.

## 1. Introduction

Flexible electronics have attracted increasing attention in recent years. Devices fabricated on thin flexible films can provide attractive features, such as the comfort of portability by rolling up or folding of the devices. Moreover, owing to their flexibility, they can be fixed on curved surfaces. With these prospects, developments pertaining to flexible displays have been energetically pursued from the early stages of flexible electronics [1,2,3,4]. In addition, development of flexible sensors has been rapidly gaining attention. For sensor applications, the convenience of flexible films that can be fixed, such as bandages, is particularly attractive. It has been demonstrated that advancements in the field of flexible sensors can lead to the realization of three-dimensionally curved surfaces capable of detecting physical and chemical phenomena, similar to human skin. Therefore, the development of flexible sensors has been the focus of several research groups, and significant progress has been made in this regard [5,6]. Furthermore, large-area applications, such as wallpapers and carpets, are another feature of flexible sensors. Sensor matrixes with large areas have been demonstrated to detect the distribution of physical quantities, such as pressure [7,8,9]. The industrialization of such large-area sensors requires an improved manufacturing process that can efficiently process the large areas. Thus, printing technology was developed to create functional layers in the atmosphere without the use of photoresists and the etching process [10,11,12]. 

In this paper, we present a novel flexible sensor that can detect wind pressure distribution. Although several reports have been presented on flexible sensors that involve physical contact between solid objects, there exists limited literature on the detection of the physical properties of wind. To the best of our knowledge, only Xu et al. have developed a thermal sensor for monitoring wind speed and direction [13]. In the present study, we achieved wind pressure detection using the strain sensor matrix developed in the flexible substrate, which was processed to be mechanically movable. We designed this new sensor based on the concept of flexible mechatronics, which is described in the next section. We believe that this concept can expand the applicability of flexible sensors based on the motion of the flexible substrate. In addition, the monitoring of wind pressure distribution demonstrated in this study is expected to contribute to various industrial domains, such as the development of automobiles and aircraft, since the control of air flow and air resistance against curved surfaces is a critical factor in these fields. We believe that the developed sensor can enables new types of aerodynamic measurements that have conventionally been difficult to perform. 

## 2. Concept of “Flexible Mechatronics”

In this work, we designed a wind pressure distribution sensor based on a new concept presented in this study: flexible mechatronics. In this concept, thin plastic films are used not only as substrates but also as mechanical moving parts. The usefulness of applying the mechanical movement of a plastic film to devices has been demonstrated in previous studies, for example, the performance improvements of piezoelectric harvesters and high-performance strain sensors capable of two-dimensional stretching [14,15,16]. In the present study, our objective was to impart three-dimensional movability to a film and use it as a sensor. To realize the concept, a suspended structure, such as a cantilever, was introduced on the film. Given the film’s thinness and low Young’s modulus, it can show mechanical displacement even when it perceives a weak load, such as wind. Furthermore, the process used to shape thin plastic films is relatively mature, and hence a suspended structure can easily be formed on the film through laser cutting or by punching with a mold. Based on this concept, we developed a wind pressure sensor using individual mechanical displacements of the shaped film and fabricated a strain sensor matrix on the film surface, as explained in the next section.

## 3. Materials and Methods

### 3.1. Fabrication of the Wind Pressure Distribution Sensor Sheet

**Figure 1:**
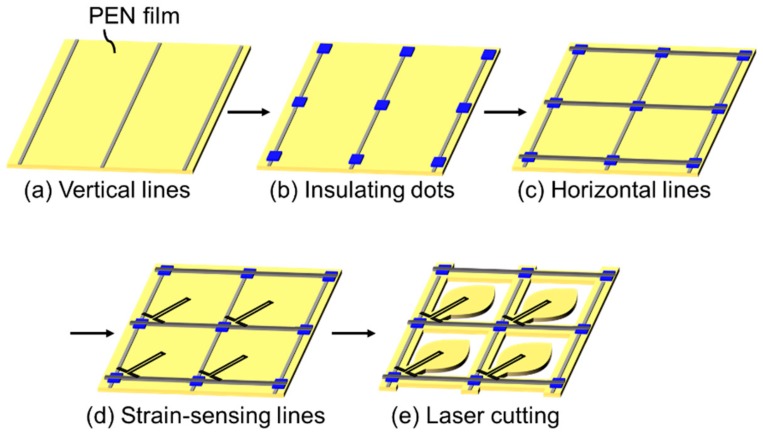
Process flow for fabricating the wind pressure distribution sensor. (**a**) Screen printing Ag paste to form vertical lines. (**b**) Screen printing UV-curable resist to form insulating dots. (**c**) Screen printing Ag paste to form horizontal lines. (**d**) Screen printing strain-sensing lines. (**e**) Laser cutting to form suspended structures.

We fabricated a sheet with a wind pressure distribution sensor by combining a resistive-strain sensor matrix and an individual moving structure composed of film substrate. The process flow is described in Figure 1a–e. To construct the strain sensor matrix, a passive matrix was fabricated by screen printing by using commercial printing equipment (Cube1515, Mino Group, Gifu, Japan). Polyethylene naphthalate (PEN; Q65HA, Toyobo Film Solutions, Tokyo, Japan), with thickness and dimensions of 50 μm and 20 mm × 20 mm, respectively, was used as the substrate. As shown in Figure 1a, a conductive Ag paste (MP-603S, Mino Group) was printed to form 13 vertical lines, each with a width of 0.5 mm, and these lines were aligned with a 10 mm pitch. The vertical lines were then cured by thermal sintering in an oven at 130 °C for 10 min. To form an insulating layer on the vertical lines, a UV-curable acryl paste (EKIRESIN MPER-9000, Goo Chemical, Kyoto, Japan) was printed into dot shapes over the vertical lines (Figure 1b). The dimension of the dot patterns was 1.5 mm × 2.5 mm, and they were aligned with a 10 mm pitch. Thirteen dots were fabricated on both the *X* and *Y* directions, resulting in a total of 169 dots. The acryl paste was cured using UV-irradiation equipment (Iwasaki Electric) with exposure of 2.0 J/cm^2^. As shown in Figure 1c, 13 horizontal lines were printed across the vertical lines under the insulating dots by using the Ag paste. Their width and pitch were equal to those of the vertical lines, with similar thermal curing conditions. At this point, the wire grid was constructed to form the passive matrix. 

Strain-sensing wires were printed, as shown in Figure 1d, to create a strain sensor matrix. Here, we used an original paste that provides a resistive film with high strain sensitivity. As the details of this paste have been reported previously [17], we only provide the outline in this paper. The paste mainly consisted of a mixture of conductive carbon black (#3030B, Mitsubishi Chemical) and polyimide vanish (IRP-1200, Sanwa Chemical) as a binder polymer. The carbon black and the polyimide varnish were mixed using an automatic hoover muller (Toyo Seiki, Seisaku-sho, Ltd., Tokyo, Japan) with a volume ratio of 6:4. The density of the carbon black was lower than that of a typical conductive paste; thus, the change in resistance against any applied strain was larger and easily detectable. The polarity of the resistance change due to a strain was such that the resistance increased as the resistive film physically expanded, similar to a general strain gauge that consists of a metal foil. The strain-sensing wire used in this study had a high gauge factor of over 50. This value was considerably higher than that of commercially available strain gauges, which have gauge factors of approximately 2. Such high sensitivity allows direct detection of changes in resistance, and hence no bridge and amp circuit were required. The resistive film was connected to the vertical and horizontal lines and was shaped as tilting–folding back lines with a width of 0.2 mm, as described in Figure 1d. The paste was thermally cured at 150 °C for 30 min. 

**Figure 2:**
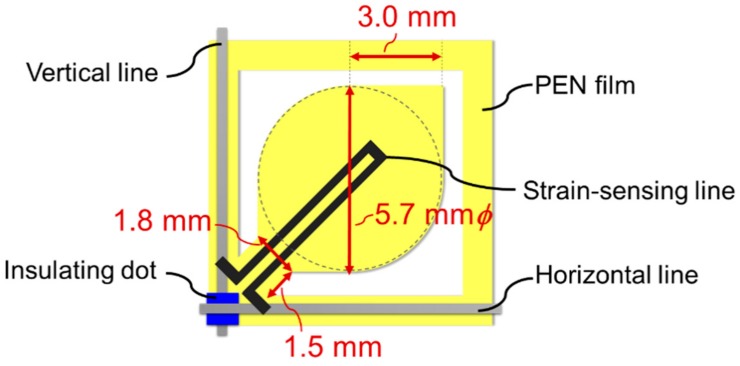
Single-element design of the wind pressure distribution sensor.

Finally, the substrate film was cut with a laser to form a suspended structure, which could be moved by wind pressure, as described in Figure 1e. In this study, the suspended structure was experimentally designed to be fan-shaped, as illustrated in Figure 2, as it needed to be easily bent by the wind. The main structure that experiences the wind was a combination of a 5.7-mm-diameter circle and a 3.0-mm-side square. In contrast, the root position was narrowed to a width of 1.8 mm and connected to the main film. The device was designed so that the fan shape could be easily displaced by the wind pressure, allowing displacement to be detected by the strain sensor matrix.

### 3.2. Evaluation of Responsivity of the Single Sensor Against Wind Pressure

**Figure 3:**
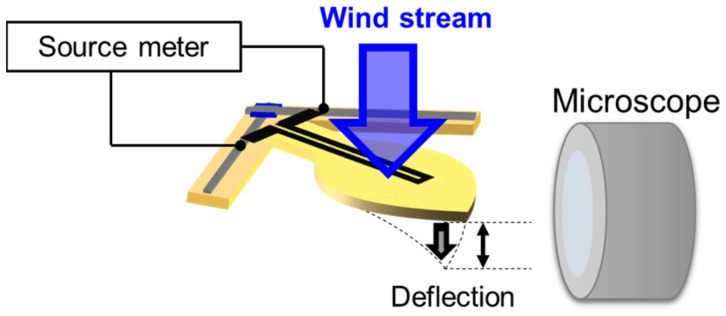
Measurement setup to evaluate the single sensor’s mechanical and electrical response against wind speed.

The responsibility of the single sensor against wind pressure was evaluated using the measurement system described in Figure 3. In this measurement, a sensor cell cut off from the fabricated sensor matrix was used to investigate the behavior of the single sensor. The vertical and horizontal lines were connected to a source meter (Keithley 2401, Cleveland, OH, USA), via electrical clips, for measuring the electrical resistance of the sensor. A microscope (VHX-6000, Keyence, Osaka, Japan) was used to observe the displacement of the suspended structure from the side. A wind stream with a speed range of 0–18 m/s was then induced using a mechanical blower (SJF-200RS-1, Suiden, Osaka, Japan) to hit the suspended structure. The wind pressure at each wind speed was estimated from Equation (1), which is provided in Section 4.2. With this setup, the change in resistance and the film displacement were observed.

### 3.3. Operation of the Wind Pressure Distribution Imaging System

**Figure 4:**
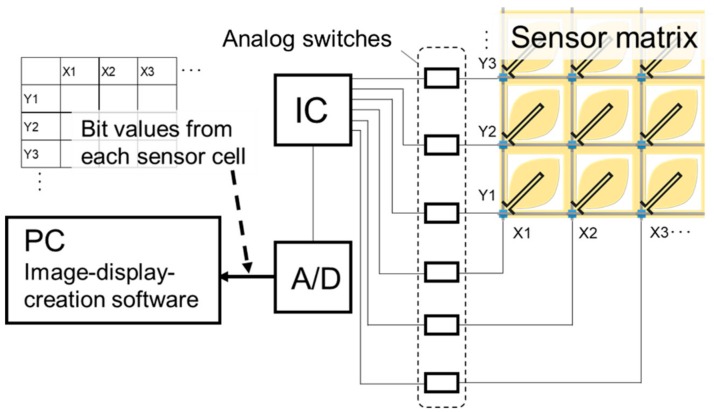
Schematic of the circuit for operating the fabricated sensor matrix.

The fabricated sensor matrix was applied to a wind pressure distribution imaging system. The system consisted of an exclusive circuit and image-display-creation software. A schematic of the circuit is shown in Figure 4. Each vertical and horizontal line in the sensor matrix was connected to an IC chip to measure the resistance of each sensor cell. The IC also controlled the analog switches, which were positioned at every pass between the lines and the IC, to determine the appropriate position for applying the voltage. The resistance of each cell was sent from the IC to an A/D converter to transform the resistance to a bit value with 256 gradations. Finally, the image-display-creation software in a PC received the bit data, before visualizing the distribution as a color tone based on the bit values and their location in the matrix. 

## 4. Results and Discussion

### 4.1. Observation of the Fabricated Sensor Matrix

**Figure 5:**
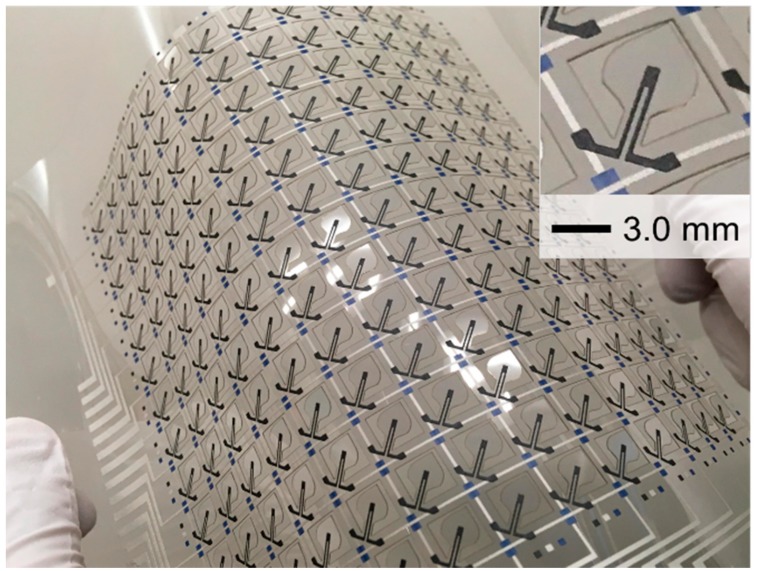
Appearance of the wind pressure distribution sensor sheet fabricated in this work.

Figure 5 shows the wind pressure distribution sensor fabricated in this study. As planned, the suspended structure was formed by laser cutting. The contour can be seen in the enlarged view in the inset. The line of the strain sensors runs along the axis of the suspended structure. The figure also shows the vertical and horizontal lines, as well as the insulating dots positioned around the cut-off regions.

The thicknesses of the vertical and horizontal lines, insulating dots, and strain sensor were measured 7.8, 21.1, 14.1, and 29.5 μm, respectively, by using a microfigure measuring instrument (ET-5000, Kosaka Laboratory, Tokyo, Japan). As these elements were designed using width in the order of submillimeters, defects, such as short circuits and disconnection, could be avoided. In other words, screen printing was confirmed to be a satisfactory method of fabricating submillimeter-order circuits of high quality.

### 4.2. Functioning of the Single Sensor Against Wind Pressure

**Figure 6:**
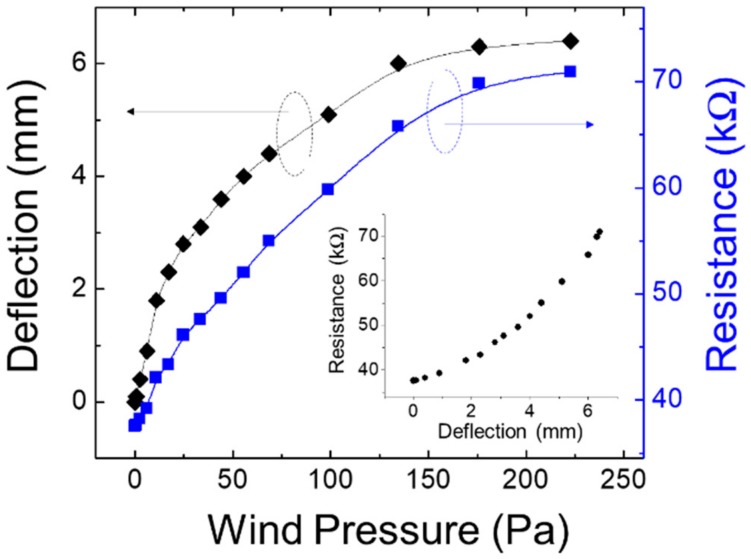
Deflection of the suspended structure and resistance recorded by the single sensor for different wind pressures.

The measurements obtained using the setup discussed in Section 3.2 are shown in Figure 6. Wind speed ranged from 0–18 m/s. Wind pressure can be calculated as:

*P* = (*ρV*^2^/2)*·C_D_*, (1)

where *P, ρ, V*, and *C_D_* correspond to wind pressure, air density, wind speed, and drag coefficient of the object exposed to the wind, respectively. Here, *C_D_* was assumed to be 1.1, a typical value used for a square and circle with a planar surface. This analysis estimated the wind pressure and load experienced by the sensor matrix. For example, since the area of the main portion of the suspended structure was 26.4 mm^2^ when it was exposed to a wind velocity of 5.0 m/s, the setup perceived 0.45 mN for a wind pressure of 17.2 Pa. As shown in Figure 6, the top of the suspended structure clearly deflects, corresponding to wind pressures below 150 Pa. The fan shape might have further emphasized the deflection of the PEN film, which had a low Young’s modulus of 2.2 GPa. For regions with pressure exceeding 150 Pa, the deflection showed a tendency toward saturation. The suspended structure was indicated to be tilted sufficiently, and its angle showed no change at this region. Although this result indicates that the responsibility of the suspended structure against the wind should be adjusted to the target wind pressure, we believe that this adjustment does not represent a major problem for the device design. In addition to using the experimental design proposed in this study, the fabricated sensor can be easily optimized by changing its structural dimensions, such as the area of the main portion of the suspended structure, width of the root portion, and thickness of the substrate film. We believe that the design concept can be widely applied to various air-flow measurements. 

The resistance of the strain sensor also changed with the deflection. As shown, its change ratio was clearly larger than that recorded, using a general strain gauge, which was made of a metal film and showed a change by only a slight percentage. Initially, it showed a resistance of 37.1 kΩ, which increased by approximately 70% at 220 Pa. The large value of the change ratio allowed for the detection at many points, as was the case with the sensor matrix fabricated in this work. The mechanical and electrical characteristics against the wind pressure described above are limited to the fabricated sensor designed experimentally in this study. It is considered that investigating the relationship between structural changes and sensor properties is an important future task.

### 4.3. Imaging of Wind Pressure Distribution Through the Developed Sensor Matrix

**Figure 7:**
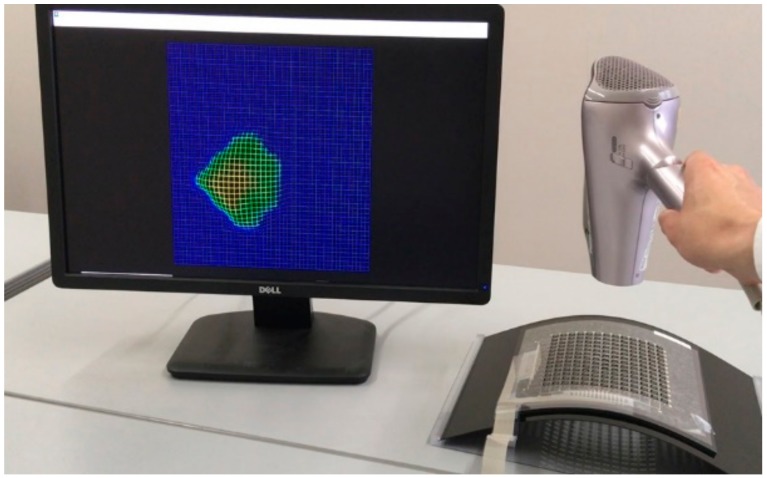
Result of wind pressure distribution imaging by using the developed sensor sheet.

The sensor matrix developed in this work was successfully operated, as shown in Figure 7. A cool wind stream was provided through a hairdryer to the sensor sheet. The wind speed ranged from 4.0–8.0 m/s. The obtained wind pressure distribution was imaged graphically by using the above-mentioned software. The result clearly showed the usefulness of the developed sensor sheet for aerodynamic evaluation, alongside Xu et al.’s recent report, which represents successful monitoring of wind speed and direction [13]. In particular, the proposed device design was specialized for the detection of wind pressure through the mechanical motion of the film. Moreover, the device configuration of the passive matrix enables it to efficiently respond to changes in the area and the points of the sensor sheet. 

As already mentioned in Section 1, this operation offers specific advantages of evaluating aerodynamic characteristics according to the degree of freedom set during the sensor installation. This method can be applied for evaluating curved surfaces, such as those of automobiles and aircraft. Furthermore, the fabrication process executed in this work is relatively suitable for fabricating large areas. Thus, it can also be applied toward imaging circulating air in living spaces, factories, and closed chambers. We consider that the addition of the mechanical function to the film substrate by using laser cutting to utilize the feature of substrate flexibility is crucial to the design. We strongly believe that this device design concept, which we call flexible mechatronics, will spur more developments in the field of flexible electronics. 

## 5. Conclusions

We developed a new sensor matrix based on the concept called flexible mechatronics to evaluate wind pressure distribution. A plastic film was used as the substrate and served the function of detecting wind pressure by shaping moving suspended structures. A strain sensor matrix was fabricated on the film surface to detect the deflection of the suspended structures via whole screen printing. We proved that the addition of mechanical functions to the substrate film effectively extends the possibilities of its application to flexible electronics.
